# Supplementary figures and images for: Identification of leaf rust resistance loci in a geographically diverse panel of wheat using genome-wide association analysis
Source: Front Plant Sci. 2023 Feb 1;14:1090163. doi: 10.3389/fpls.2023.1090163 (PMC9929074; doi:10.3389/fpls.2023.1090163)

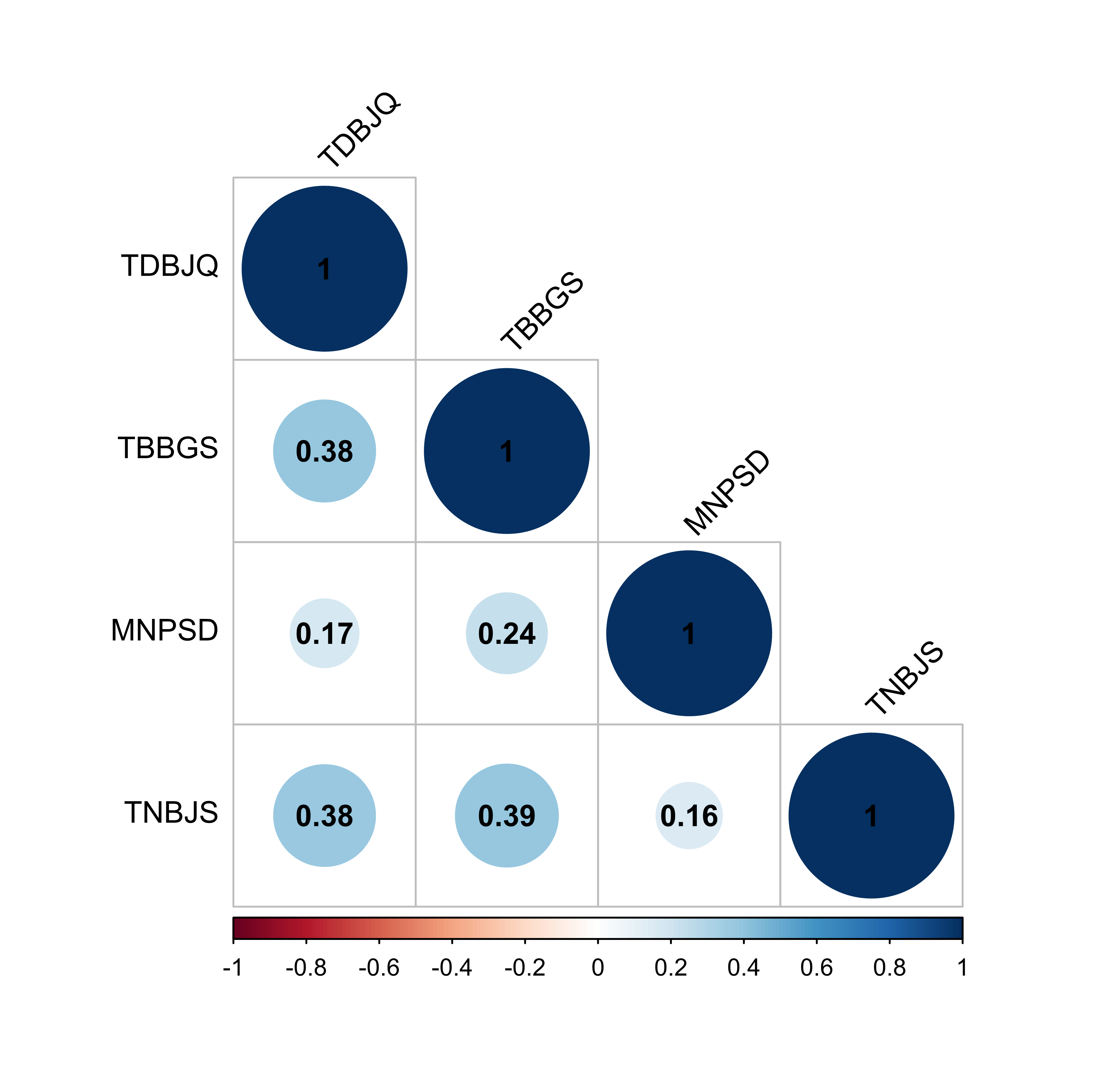

Supplement: Supplementary Figure S1 — Correlation analysis among the phenotypic data of 365 wheat accessions evaluated for their reaction to P. triticina races TDBJQ, TBBGS, MNPSD, and TNBJS. [file Image_1.tif]

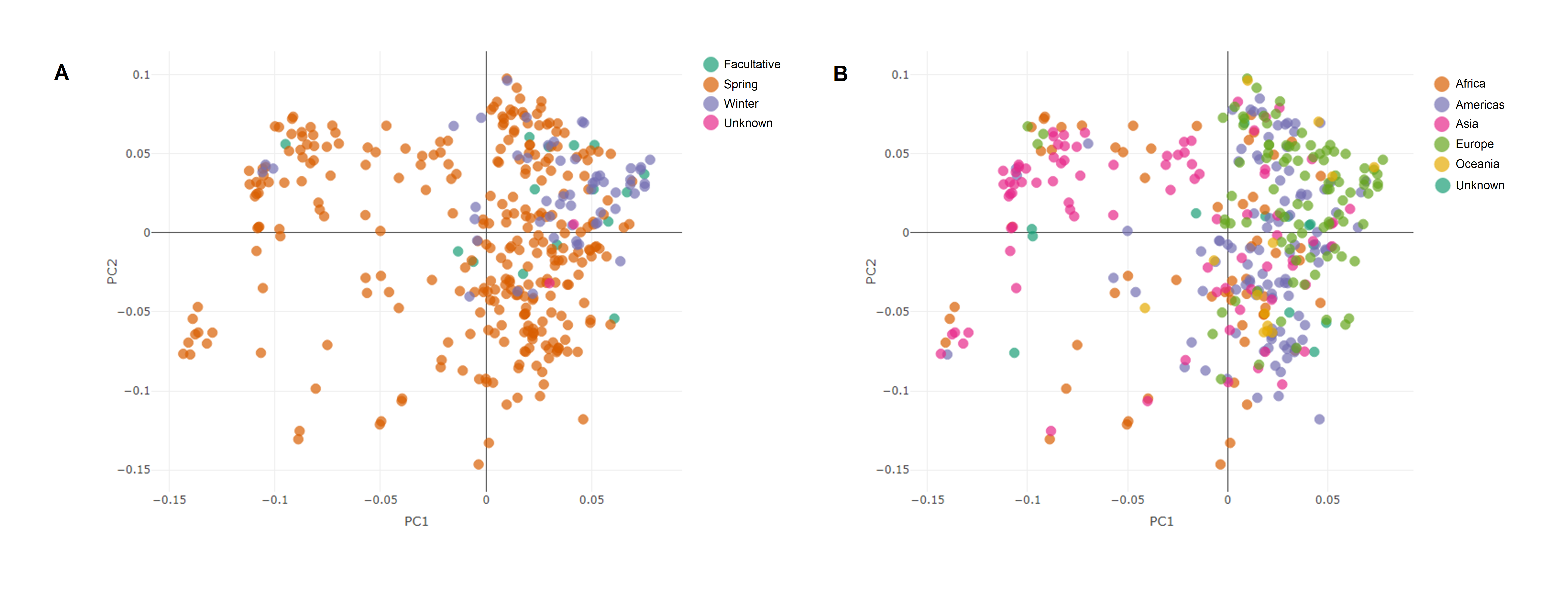

Supplement: Supplementary Figure S2 — Scatterplot based on the first two components (PC1 and PC2) from PCA for (A) growth habit and (B) geographic origin. [file Image_2.tif]

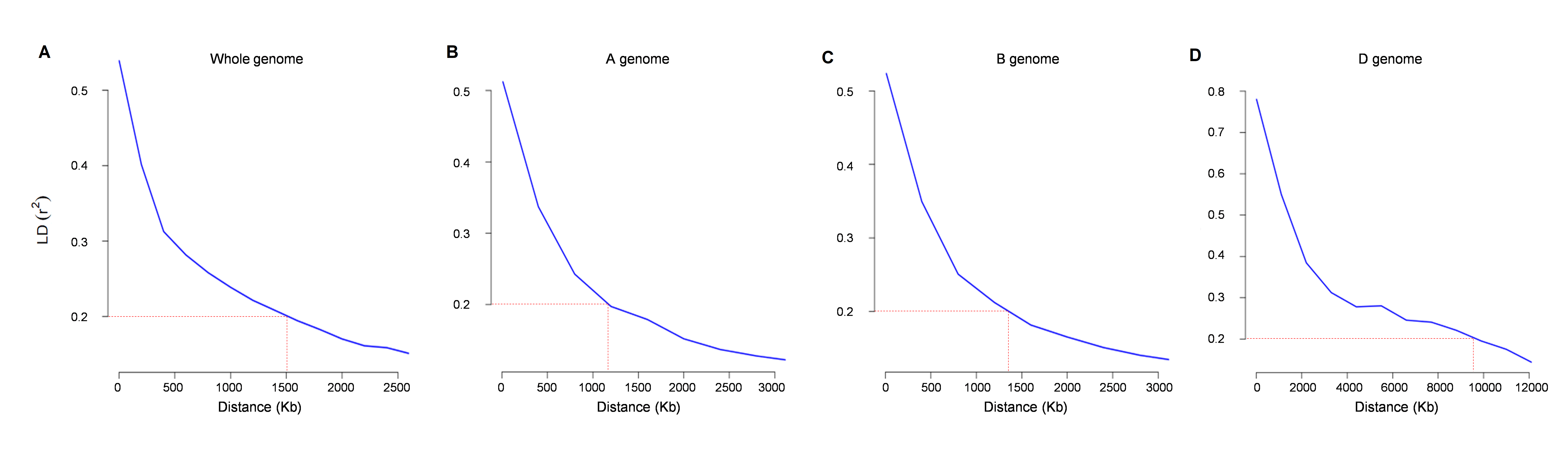

Supplement: Supplementary Figure S3 — Intra-chromosomal linkage disequilibrium in the diversity panel for (A) whole genome and, individually in the (B) A, (C) B, and (D) D sub-genomes. [file Image_3.tif]

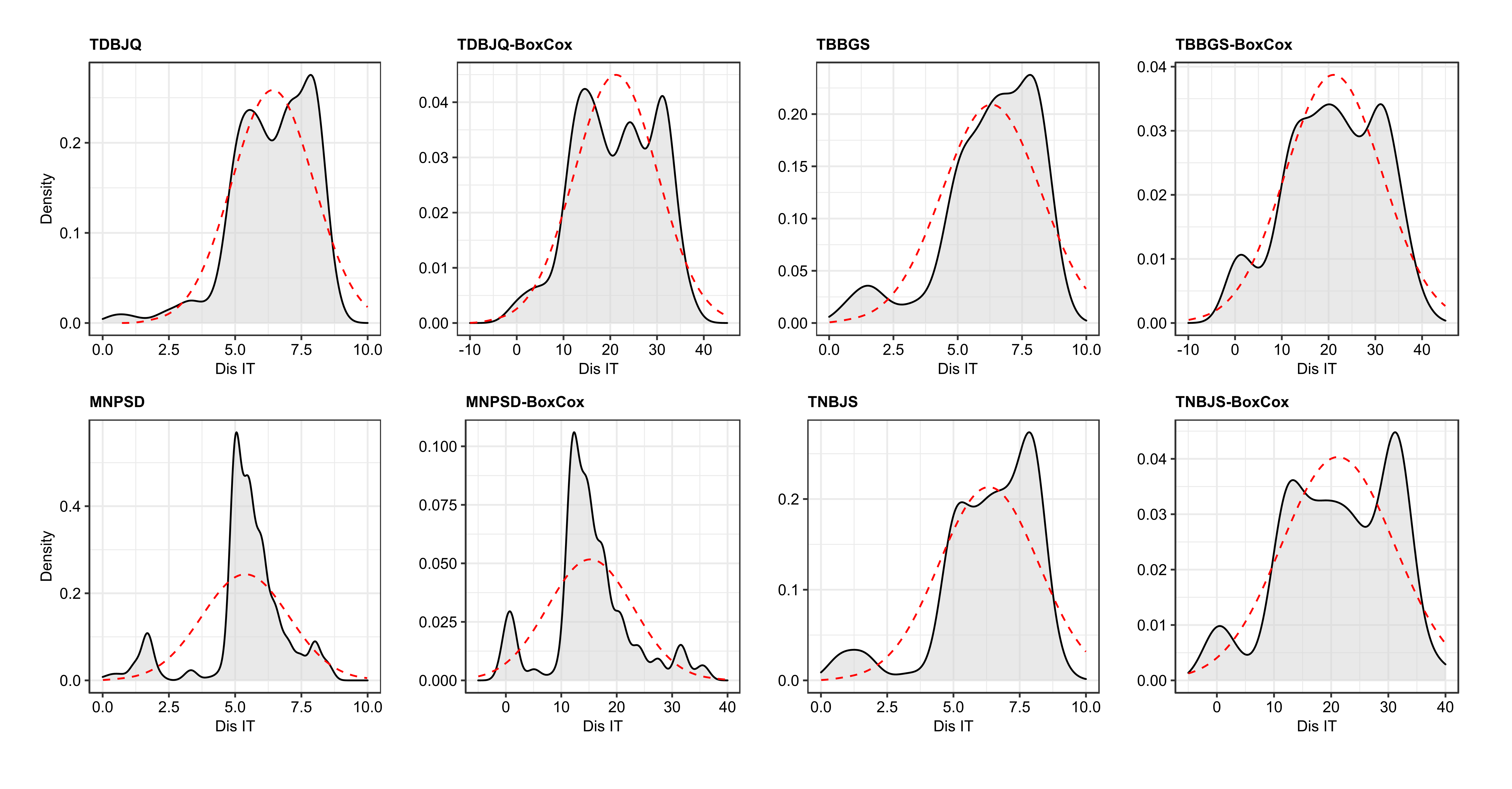

Supplement: Supplementary Figure S4 — Box-cox transformations performed for Pt races TDBJQ, TBBGS, MNPSD, and TNBJS to normalize the data. [file Image_4.tif]
